# The Role of Na^+^/K^+^-ATPase during Chick Skeletal Myogenesis

**DOI:** 10.1371/journal.pone.0120940

**Published:** 2015-03-16

**Authors:** Taissa Neustadt Oliveira, Ana Claudia Possidonio, Carolina Pontes Soares, Rodrigo Ayres, Manoel Luis Costa, Luis Eduardo Menezes Quintas, Cláudia Mermelstein

**Affiliations:** 1 Laboratório de Diferenciação Muscular e Citoesqueleto, Instituto de Ciências Biomédicas, Universidade Federal do Rio de Janeiro, Rio de Janeiro, Brazil; 2 Laboratório de Farmacologia Bioquímica e Molecular, Instituto de Ciências Biomédicas, Universidade Federal do Rio de Janeiro, Rio de Janeiro, Brazil; Memrial University, CANADA

## Abstract

The formation of a vertebrate skeletal muscle fiber involves a series of sequential and interdependent events that occurs during embryogenesis. One of these events is myoblast fusion which has been widely studied, yet not completely understood. It was previously shown that during myoblast fusion there is an increase in the expression of Na^+^/K^+^-ATPase. This fact prompted us to search for a role of the enzyme during chick *in vitro* skeletal myogenesis. Chick myogenic cells were treated with the Na^+^/K^+^-ATPase inhibitor ouabain in four different concentrations (0.01-10 μM) and analyzed. Our results show that 0.01, 0.1 and 1 μM ouabain did not induce changes in cell viability, whereas 10 μM induced a 45% decrease. We also observed a reduction in the number and thickness of multinucleated myotubes and a decrease in the number of myoblasts after 10 μM ouabain treatment. We tested the involvement of MEK-ERK and p38 signaling pathways in the ouabain-induced effects during myogenesis, since both pathways have been associated with Na^+^/K^+^-ATPase. The MEK-ERK inhibitor U0126 alone did not alter cell viability and did not change ouabain effect. The p38 inhibitor SB202190 alone or together with 10 μM ouabain did not alter cell viability. Our results show that the 10 μM ouabain effects in myofiber formation do not involve the MEK-ERK or the p38 signaling pathways, and therefore are probably related to the pump activity function of the Na^+^/K^+^-ATPase.

## Introduction

Muscle fibers are multinucleated cells that have a highly organized myofibrillar cytoskeleton that enables them to be extremely efficient in contraction. The formation of skeletal muscle fibers involves a series of sequential events that begins, during embryogenesis, with the commitment of mononucleated myoblasts and culminates with cell fusion. The formation of long and striated multinucleated myotubes depends on myoblast recognition and fusion. Prior to fusion, myoblasts undergo a number of biochemical and morphological changes, particularly in their plasma membrane, that enables them to fuse. These changes include the expression and spatial organization of membrane proteins and lipids [1–3]. For instance, cholesterol depletion by methyl-β-cyclodextrin enhances myoblast fusion [4]. Other studies showed a decrease in the concentration of cholesterol in the membrane of fusing myoblasts, and this decrease was related to an increasing in membrane fluidity that is necessary for fusion [5].

The Na^+^/K^+^-ATPase enzyme is an essential component of the plasma membrane of all animal cells. This enzyme is responsible for the transport of Na^+^ and K^+^ ions across the plasma membrane against their electrochemical gradients, and helps the maintenance of membrane potential. The Na^+^/K^+^-ATPase is composed by two subunits, α and β. The main subunit, α, also known as catalytic or functional, has 4 isoforms in mammals; whereas all cells express the housekeeping isoform α 1, the others have a more restrict tissue distribution. Rat skeletal muscle *in vivo* expresses also α 2, which is the most abundant isoform [6–8] as well as mouse C2C12 cells [9,10]. On the other hand, rat skeletal muscle primary cultures only express α 1 [11,12], which could be due to the lack of innervation [13], and a similar profile occurs in L6 and L8 rat myogenic cell lines [14]. In primary cultures of chick skeletal muscle cells, one isoform has been detected so far [15,16]. An increase in the activity and expression of Na^+^/K^+^-ATPase has been shown during chick myogenesis using different techniques [15,17–19]. Interestingly, intracellular Na^+^ concentration augments during murine myoblast fusion [20], suggesting that the upregulation of Na^+^ pumps during chick myogenesis may be a response to increased Na^+^ load [16,19]. Inhibition of Na^+^/K^+^-ATPase has been an important strategy to study the role of the enzyme during muscle differentiation. Experimentally, the most widely used Na^+^/K^+^-ATPase inhibitor is ouabain. Ouabain is a potent cardiotonic steroid obtained from mature African seeds of *Strophantus gratus* and *Acokanthera ouabaio* plants. Interestingly, recent studies indicate the possible endogenous synthesis of ouabain-like steroids in mammalian tissues. In 1991, an isomer of ouabain was identified as an endogenous hormone synthesized by the adrenal gland and also by the hypothalamus, but its mechanism of action and physiological significance have not yet been precisely determined [21,22].

Although the effect of cardiotonic steroids in chick skeletal myogenesis is unknown, previous studies demonstrated that addition of high concentrations (300–400 μM) of ouabain to L6 or C2 myoblast cell line produced nearly complete inhibition of myoblast fusion, and removal of ouabain allowed complete fusion to occur [23]. Nevertheless, an overall reduction rate of protein synthesis was considered to be a consequence of ouabain-induced dissipation of Na^+^ and K^+^ gradients and low rates of cell fusion, i.e., a nonspecific role of Na^+^/K^+^-ATPase in the phenomenon [20]. In recent years, however, it has been discovered that Na^+^/K^+^-ATPase also mediates the activation of signaling cascades through protein-protein interaction, such as the MEK-ERK and the p38 pathways, upon binding of cardiotonic steroids [24,25]. These novel noncanonical Na^+^/K^+^-ATPase functions, which are responsible for different cellular effects including cell growth and differentiation, are assumed to be carried out by a pool of nonpumping Na^+^ pumps localized in caveolae, cholesterol-enriched invaginations of the plasma membrane [24,26]. For instance, the lack of caveolae promoted by cholesterol depletion or caveolin-1 depletion by siRNA abrogates ouabain-evoked signaling [27,28]. Moreover, Na^+^/K^+^-ATPase is involved in controlling the trafficking of caveolin-1 and cholesterol distribution, suggesting that it is important for caveolar assembly [29,30].

Our group has demonstrated that cholesterol depletion exacerbates chick myoblast fusion [4,31] and cell proliferation [32]. Considering that cholesterol and caveolae are important partners for Na^+^/K^+^-ATPase function, the aim of this study was to investigate the possible role of the Na^+^/K^+^-ATPase during the initial steps of chick skeletal muscle differentiation using ouabain as a tool.

## Materials and Methods

### Antibodies and fluorescent probes

DNA-binding probe DAPI (4,6-Diamino-2-phenylindole dihydrochloride) was purchased from Molecular Probes (USA). Rabbit polyclonal anti-desmin was from Sigma Chemical Co. (USA). Alexa Fluor 546-goat anti-rabbit IgG antibody was from Molecular Probes (USA).

### Primary myogenic cell cultures

This study using chick embryos was approved by the Ethics Committee for Animal Care and Use in Scientific Research from the Federal University of Rio de Janeiro and received the approval number: DAHEICB 004. All cell culture reagents were purchased from Invitrogen (São Paulo, Brazil). Primary cultures of myogenic cells were prepared from breast muscles of 11-day-old chick embryos [4]. Cells were plated at an initial density of 7.5 x 10^5^ cells/35 mm culture dishes onto 22 mm-Aclar plastic coverslips (Pro-Plastics Inc., USA) previously coated with rat tail collagen. Cells were grown in 2 ml of medium (minimum essential medium with the addition of 10% horse serum, 0.05% chick embryo extract, 1% L-glutamine and 1% penicillin-streptomycin) under humidified 5% CO_2_ atmosphere at 37°C.

The percentage of myoblasts in these cell cultures was calculated by the double-labeling of 24-hour cultures with both DAPI (nuclear staining) and anti-desmin antibody (applied herein to define a muscle-specific marker) and subsequently counting the number of desmin-positive cells out of the total number of cells in the field. On average, myoblasts made up 80% of each culture and nonmyogenic cells comprised 20%.

Twenty-four-hour cultures were treated with ouabain at a final concentration of 0.01 μM, 0.1 μM, 1 μM or 10 μM, or with the MEK-ERK inhibitor U0126 (Sigma Chemical Co., USA) at a final concentration of 10 μM or with the p38 inhibitor SB202190 (Tocris Bioscience, USA) at a final concentration of 5 μM. 24 hours after treatment, cultures were washed with fresh cultured medium and grown for the next 24 or 48 hours.

Some 24-hour cultures were treated with the cholesterol depleting agent methyl-β-cyclodextrin (MβCD, Sigma Chemical Co., USA) at a final concentration of 2 mM for 30 min. The 2 mM final concentration of MβCD was chosen for cell culture treatments because our group has previously shown that 2 mM of MβCD is sufficient to induce skeletal muscle cell differentiation without interfering with cell viability [4,32]. After MβCD treatment, cultures were washed with fresh cultured medium and either treated with ouabain 10 μM for or grown in normal culture medium for the next 48 hours.

### Immunofluorescence microscopy and digital image acquisition

Cells were rinsed with PBS and fixed with 4% paraformaldehyde in PBS for 10 min at room temperature. Cells were then permeabilized with 0.5% Triton-X 100 in PBS for 30 min. The same solution was used for all subsequent washing steps. Cells were incubated with primary antibodies for 1 h at 37°C. After incubation, cells were washed for 30 min with 0.5% Triton-X 100 in PBS and incubated with secondary antibodies for 1 h at 37°C. The cells were washed again for 30 min with 0.5% Triton-X 100 in PBS and once with 0.9% NaCl. Nuclei were labeled with DAPI (0.1 μg/ml in 0.9% NaCl) for 5 min. Cells were mounted in ProLong Gold antifade reagent (Molecular Probes, USA) and examined with an Axiovert 100 microscope (Carl Zeiss, Germany). Images were acquired with a C2400i integrated charge-coupled device camera (Hamamatsu Photonics, Shizuoka, Japan) and an Argus 20 image processor (Hamamatsu Photonics, Japan). Control experiments with no primary antibodies showed only faint background staining.

### Quantification of cell cultures

Cultures (untreated and treated) were fixed and double labeled for desmin and the nuclear dye DAPI and merged images were used for checking the presence of nuclei within mononucleated or multinucleated cells. Nuclei (from mononucleated and multinucleated cells) were counted in fifty randomly chosen microscope fields (3 culture dishes, 50 fields in each dish) at a magnification of x 400.

Myotube thickness was measured in the thicker region of each multinucleated myotube, for all myotubes in each microscopic field, in at least 50 different fields for each experimental condition.

The number of myotubes was counted in at least 50 different fields for each experimental condition.

The quantification of the number of myoblasts and fibroblasts was manually performed in chick myogenic cells immunolabeled for desmin (muscle-specific marker) and the nuclear dye DAPI. The DAPI labeling enables the identification of these two cell types by their nuclear morphologies and fluorescence intensities. In DAPI-labeled chick myogenic cultures, muscle fibroblasts have large, flattened and pale nuclei whereas myoblasts have small, round and bright nuclei. We also performed an automated quantification of nuclei area in which we could analyze the differences between the distributions of size categories (fibroblasts are larger than myoblasts) in both untreated and ouabain-treated condition. In both methodologies (manual and automated) we achieved similar results.

All data were collected from three independent experiments. All quantifications were performed using the public domain software ImageJ (http://rsb.info.nih.gov/ij/).

### Cell viability

Chick myogenic cells were plated at an initial density of 2 x 10^4^ cells per well in 96-well plates. Cells were grown in 2 ml of medium (minimum essential medium with the addition of 10% horse serum, 0.05% chick embryo extract, 1% L-glutamine and 1% penicillin-streptomycin) under humidified 5% CO_2_ atmosphere at 37°C. Twenty-four-hour cultures were treated with ouabain at a final concentration of 0.01 μM, 0.1 μM, 1 μM or 10 μM, or with the MEK-ERK inhibitor U0126 at a final concentration of 10 μM, or with the p38 inhibitor SB202190 at a final concentration of 5 μM. 24 hours after treatment, cultures were washed with fresh cultured medium and cell viability was measured by the XTT assay [33]. XTT 2,3-bis(2-methoxy-4-nitro-5-sulfophenyl)-5-[(phenylamino) carbonyl]-2//-tetrazolium hydroxide, Sigma Chemical Co., USA is metabolically reduced in viable cells to a water-soluble formazan product. This reagent allows direct absorbance readings. XTT was prepared at 1 mg/ml in prewarmed (37°C) MEM without serum. PMS (phenazine methosulfate, Sigma Chemical Co., USA) was prepared at 5 mM (1.53 mg/ml) in phosphate buffered solution (PBS). Fresh XTT and PMS were mixed together in the appropriate concentrations. For a 0.025 mM PMS-XTT solution, 25 μl of the stock 5 mM PMS was added per 5 ml of XTT (1 mg/ml). One hundred μl of this mixture (final concentration, 50 μg XTT and 0.38 μg PMS per well) was added to each 96-well containing chick myogenic cell cultures. After incubation at 37°C for 24 h, the absorbance at 492 nm was measured with a SUNRISE-Basic Tecan spectrophotometer (Austria).

### Statistical analysis

All the values were represented as the means ± standard error. Statistical analysis was performed with one-way ANOVA on Ranks with Newman-Keuls Post Hoc test and statistical significance was defined as p<0.05.

## Results and Discussion

In this study we examined the role of Na^+^/K^+^-ATPase during skeletal muscle differentiation. We used chick myogenic cell cultures to probe the involvement of Na^+^/K^+^-ATPase in the initial steps of myogenesis. In order to modulate Na^+^/K^+^-ATPase activity, cells were treated with different concentrations (0.01–10 μM) of the cardiotonic steroid ouabain. We also analyzed the involvement of MEK-ERK and p38 signaling pathways in the ouabain-induced effects by using the MEK-ERK inhibitor U0126 and the p38 inhibitor SB202190. The chick myoblast primary culture is a robust *in vitro* model of myogenesis. Skeletal myogenesis proceeds in these cell cultures through the following main sequential stages: myoblast proliferation, cell cycle withdrawal, myoblast alignment and fusion, and their subsequent differentiation into striated multinucleated myotubes. The fact that these steps have been well characterized in the last 50 years makes this a valuable model for the study of the role of Na^+^/K^+^-ATPase during skeletal muscle differentiation.

**Fig 1:**
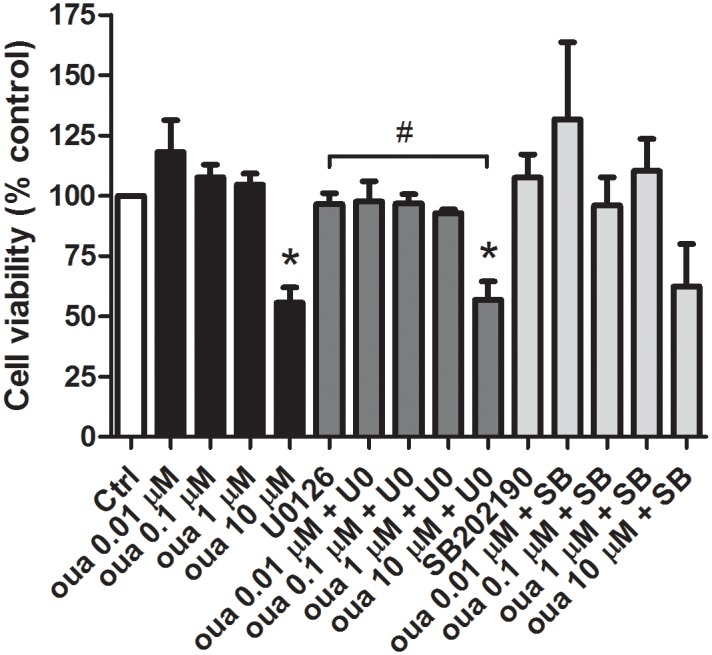
Effects of ouabain in cell viability. Chick myogenic cells were grown for 24 h and treated with different concentrations of ouabain for 24 h. A MTT-based method was used to analyze cell viability in untreated and ouabain-treated cells. Data represent mean absorbance minus background from triplicate wells. *p<0.05 compared to untreated cells (Ctrl) and ^#^p<0.05 for comparison to the respective control; one-way ANOVA on Ranks with Newman-Keuls Post Hoc test; n = 3.

First we tested the viability of cells treated with ouabain, or the MEK-ERK inhibitor U0126, or the p38 inhibitor SB202190. Experiments were done with untreated and treated myogenic cell cultures using a XTT-based cell viability method [33]. Our results show that 0.01, 0.1 and 1 μM ouabain did not induce changes in cell viability (Fig. 1). The highest concentration tested, 10 μM, reduced viability by 45%. This effect is consistent with affinity of ouabain to chick muscle Na^+^/K^+^-ATPase (Kd = 0.5–2 μM) [15,18]. Indeed, 10 μM ouabain would inhibit bulk cellular Na^+^/K^+^-ATPase activity almost completely [18]. In all concentrations of ouabain tested, inhibition of MEK, and probably p38 (p = 0.088 compared to SB202190 alone) had no effect (Fig. 1). It is important to notice that, at the concentrations tested, the MEK-ERK inhibitor U0126 and the p38 inhibitor SB202190 alone did not induce changes in cell viability in chick myogenic cells (Fig. 1).

**Fig 2:**
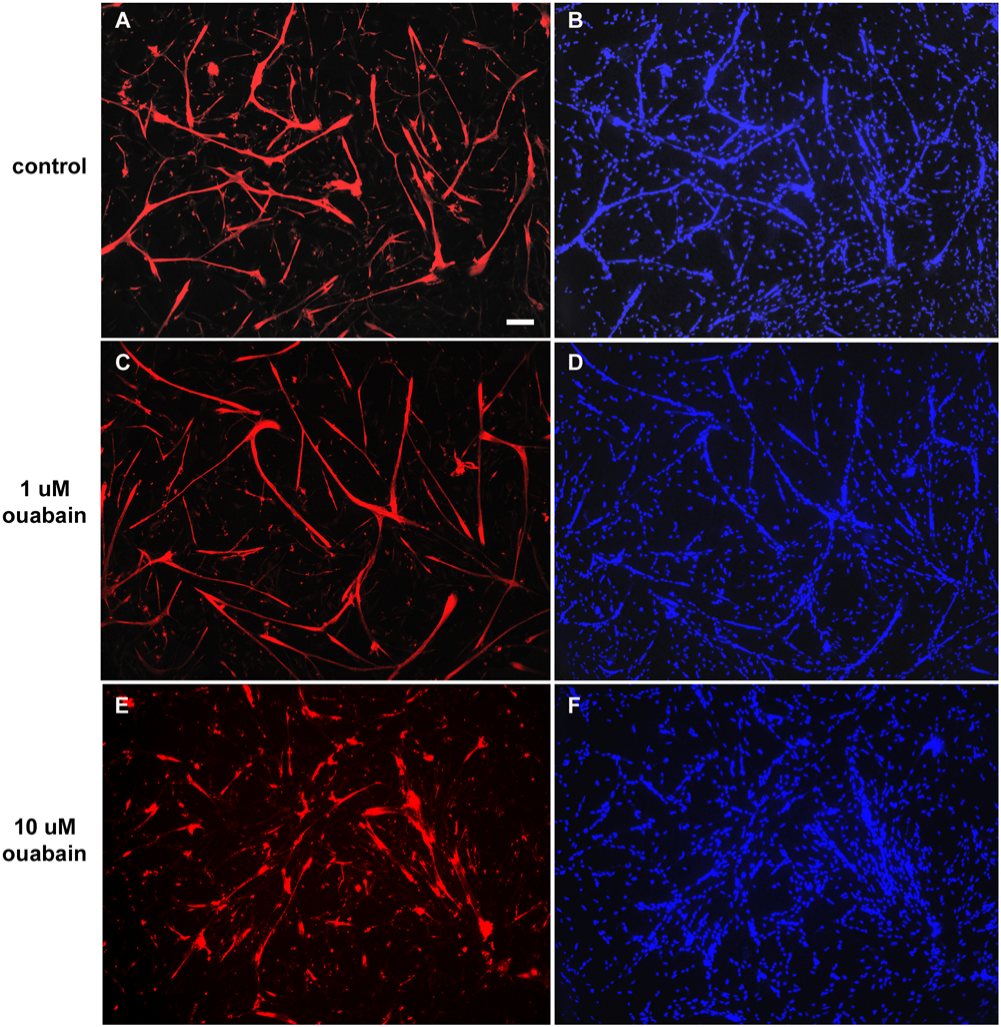
Effects of ouabain in myogenic differentiation. Chick myogenic cells were grown for 24 h, treated with 1 or 10 μM ouabain and fixed after 24 h. Cells were immunostained desmin (red) and DAPI (blue). Three independent experiments were performed and one representative image of each culture condition is shown. Scale bar = 100 μm.

**Fig 3:**
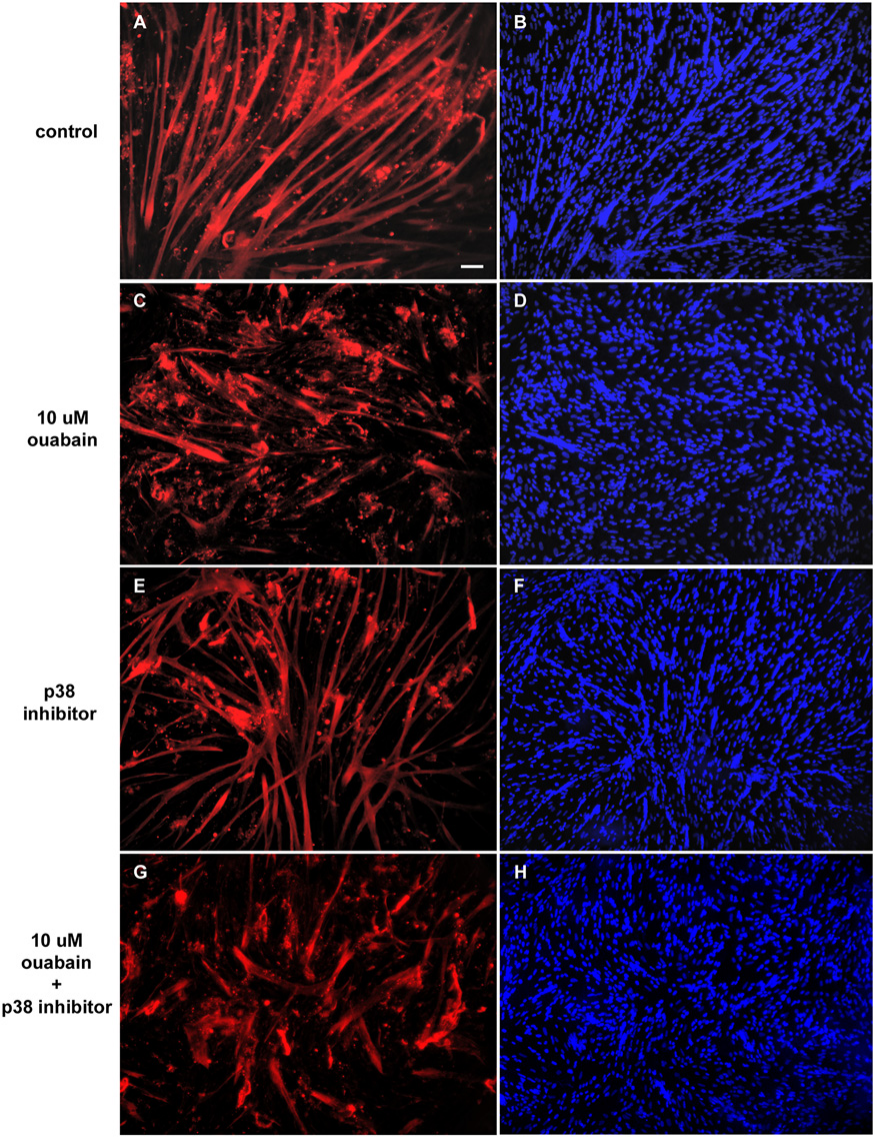
Effects of p38 pathway in ouabain-induced effects in myogenic differentiation. Chick myogenic cells were grown for 24 h, treated with 10 μM ouabain and/or the p38 inhibitor SB202190 and fixed after 24 h. Cells were immunostained desmin (red) and DAPI (blue). Three independent experiments were performed and one representative image of each culture condition is shown. Scale bar = 50 μm.

**Fig 4:**
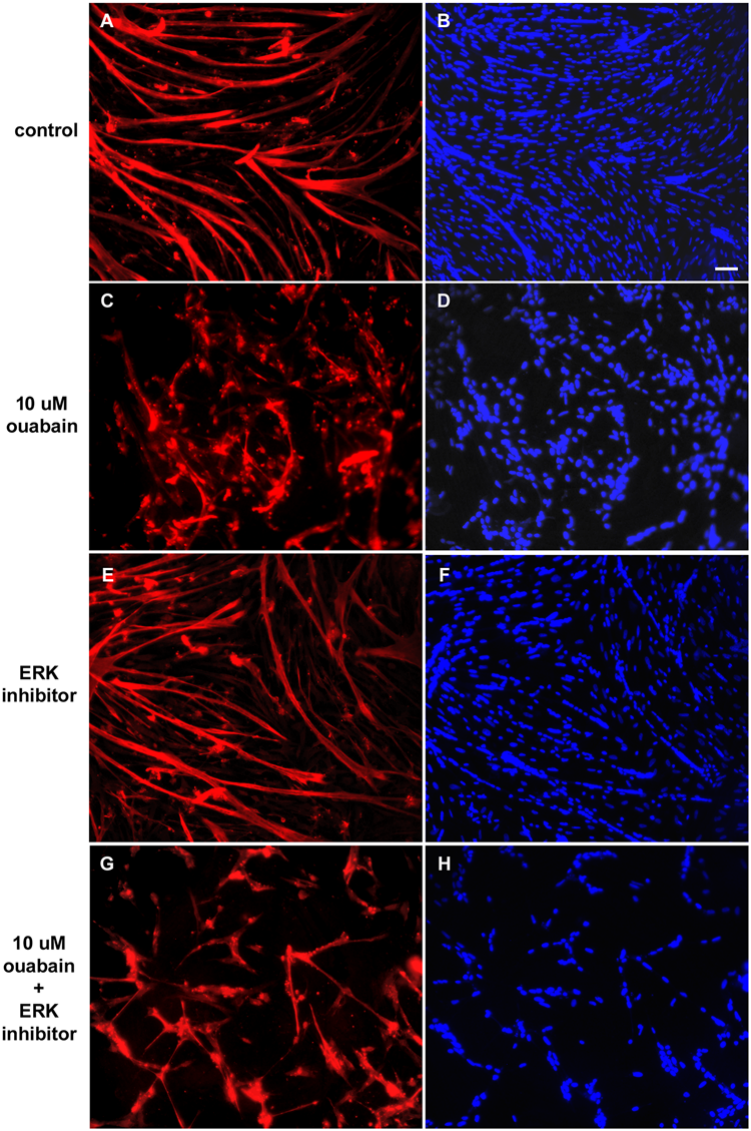
Effects of MEK-ERK pathway in ouabain-induced effects in myogenic differentiation. Chick myogenic cells were grown for 24 h, treated with 10 μM ouabain and/or the MEK-ERK inhibitor U0126 and fixed after 24 h. Cells were immunostained desmin (red) and DAPI (blue). Three independent experiments were performed and one representative image of each culture condition is shown. Scale bar = 50 μm.

**Fig 5:**
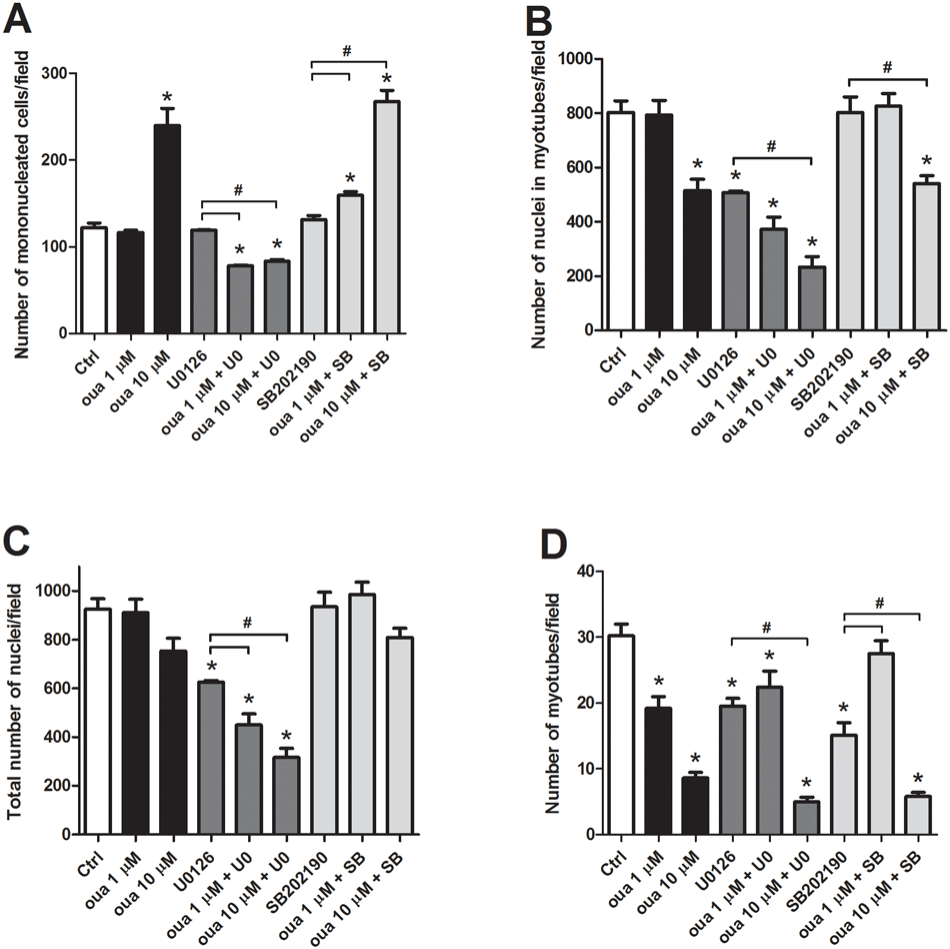
Quantification of ouabain effect in mononucleated and multinucleated myogenic cells. The number of mononucleated cells (A), the number of nuclei within myotubes (B), the total number of nuclei (C), as well as the number of myotubes (D) per field was quantified in untreated and treated cultures (from images represented in Figs. 2–4). *p<0.05 compared to untreated cells (Ctrl) and ^#^p<0.05 for comparison to the respective control; one-way ANOVA on Ranks with Newman-Keuls Post Hoc test; n = 3. At least 50 microscopic fields for each culture condition were scored in at least three independent experiments.

Next, we investigated the influence of ouabain (Fig. 2), the p38 inhibitor SB202190 (Fig. 3) and the MEK-ERK inhibitor U0126 (Fig. 4) in skeletal muscle differentiation. Immunofluorescence images of two-day cultured primary chick skeletal muscle cells show that 10 μM ouabain clearly inhibited myogenic differentiation, which can be seen by a reduction in the formation of multinucleated myotubes (compare Figs. 2a-b with Figs. 2e-f). In contrast, 1 μM ouabain showed no effect in the formation of myotubes as compared to untreated cultures (compare Figs. 2a-b with Figs. 2c-d). Interestingly, the quantification of the DAPI nuclear staining showed an increase in the number of isolated mononucleated cells and a decrease in the number of nuclei within multinucleated myotubes in cultures treated with 10 μM ouabain (Figs. 5a and 5b). This disturbance in myotube formation suggests that myoblast fusion could be compromised. Indeed, the number of myotubes is lower with 10 μM ouabain (Fig. 5d). Similarly, in rodent L6 or C2 myoblasts, ouabain (> 300 μM) hampers myogenesis [23]. The striking difference in the concentration to achieve this effect between species is possibly due to existence of the ouabain-resistant α1 isoform in rodents [34]. Increase in the expression of Na^+^/K^+^-ATPase by heterologous transfection has been shown to enhance myoblast fusion in the L8 rat myogenic cell line [14]. Nevertheless, an overall reduction rate of protein synthesis was considered to be a consequence of ouabain-induced dissipation of Na^+^ and K^+^ gradients and low rates of cell fusion, i.e., a nonspecific role of Na^+^/K^+^-ATPase in the phenomenon [20]. We also tested a lower concentration of ouabain (1 μM) and no change in the number of mononucleated cells (Fig. 5a) and in the number of nuclei within myotube and in the total number of nuclei (Fig. 5b and 5c, respectively) was observed, although the number of myotubes was reduced (Fig. 5d). These results show that different concentrations of ouabain can induce different effects in skeletal muscle fiber formation.

Our results show that the p38 inhibitor alone had no effect in the formation of myotubes (Figs. 3 and 5a-c). In mammals, p38 was shown to be a key kinase during myogenesis [35], involved in the initiation of differentiation [36], stimulating MEF2-MyoD factors in the initial steps of muscle differentiation [37]. In fact, Lee and colleagues (2002) have demonstrated that p38 inhibits ERK1/2 pathway stopping proliferation in order to start myotube development [38]. In contrast, the role of p38 in avian skeletal myogenesis is poorly understood, but it was suggested in v-Src transformed chicken myoblasts that p38 would have a similar growth-inhibiting and differentiation-inducing function [39]. Our work reveals that p38 probably has a minor influence in the initial steps of chick myogenesis. Moreover, considering that most effects of SB202190 plus 10 μM ouabain had a similar profile to ouabain alone (Fig. 5), this strongly suggests that the p38 pathway is not involved in the inhibition of myogenesis caused by ouabain.

Interestingly, our data show that the ERK inhibitor alone induced a reduction in the formation of myotubes (Figs. 4 and 5b) as well as in the total number of nuclei and in the number of myotubes in these cultures (Figs. 5c and 5d, respectively). In fact, in mammalian skeletal myocytes, ERK1/2 have been shown to be important in cell proliferation [40] and in the terminal stage of muscle differentiation [41,42]. Some reports in chick point to a role of ERK1/2 in skeletal muscle development [43,44], but this is the first time that a direct effect of ERK1/2 is demonstrated during chick embryo skeletal muscle differentiation. Further, 1 or 10 μM ouabain potentiated these effects (Fig. 5). Therefore, as U0126 did not antagonize the ouabain effect, except in the case of the number of mononucleated cells at 10 μM ouabain (Fig. 5a), we believe that ERK1/2 signaling is probably not involved in the effect of ouabain in myogenesis.

The collection of results presented above show that 10 μM ouabain inhibits the formation of multinucleated myotubes and that neither the MEK-ERK nor the p38 inhibitors could impede this effect (Figs. 2–5).

**Fig 6:**
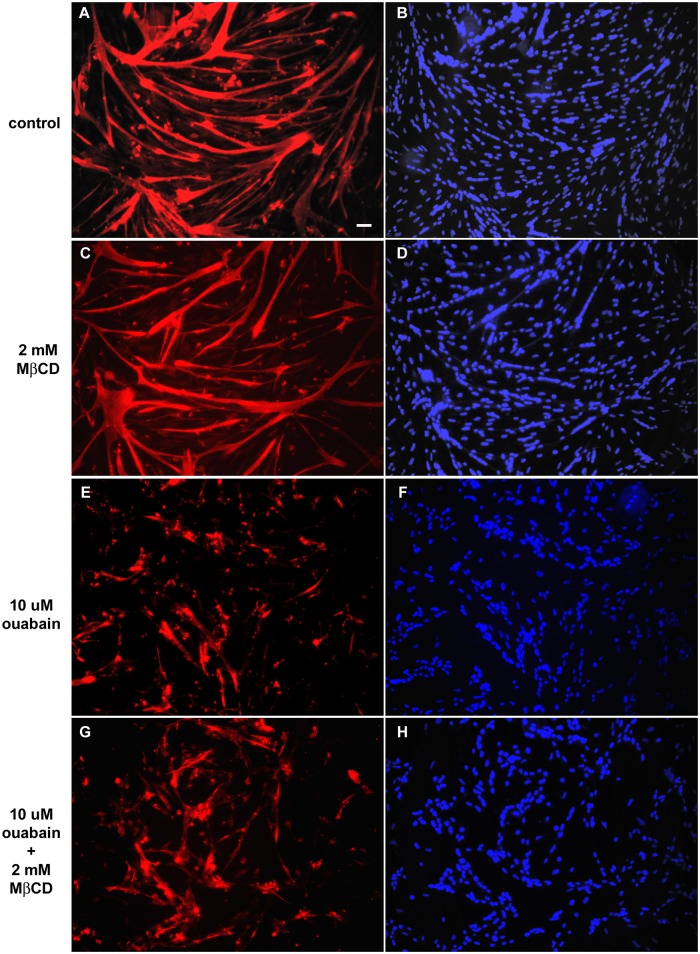
Effects of methyl-β-cyclodextrin (MβCD) on ouabain-induced effects in myogenic cells. Chick myogenic cells were grown for 24 h, treated with 2 mM MβCD for 30 min and/or 10 μM ouabain for 24 h. After 24 h cells were immunostained for desmin (red) and DAPI (blue). Three independent experiments were performed and one representative image of each culture condition is shown. Scale bar = 40 μm.

**Fig 7:**
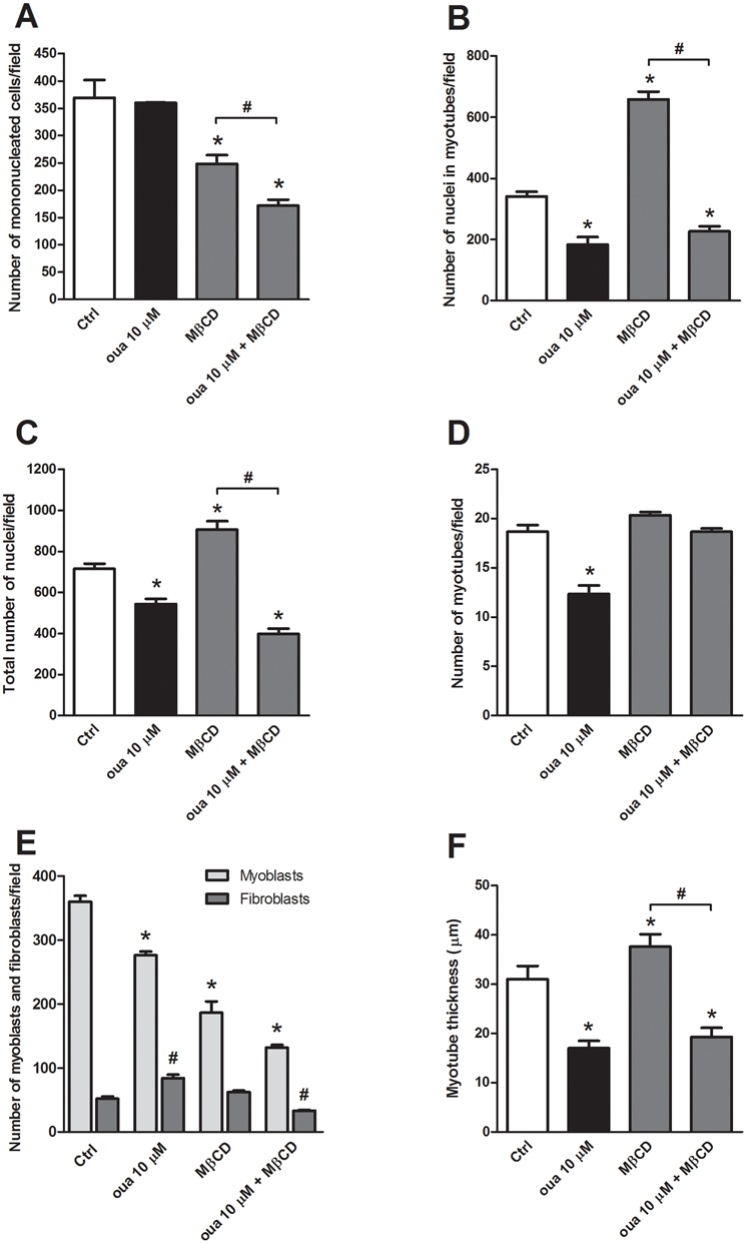
Quantification of methyl-β-cyclodextrin effect (MβCD) on ouabain-treated mononucleated and multinucleated myogenic cells. The number of mononucleated cells (A), the number of nuclei within myotubes (B), the total number of nuclei (C), the number of myotubes (D), the number of myoblasts and fibroblasts (E), as well as myotube thickness (F) was quantified in untreated and treated cultures (from images represented in Fig. 6). *p<0.05 compared to untreated myoblasts (Ctrl) and ^#^p<0.05 for comparison to untreated fibroblasts (Ctrl); one-way ANOVA on Ranks with Newman-Keuls Post Hoc test; n = 3. At least 50 microscopic fields for each culture condition were scored in at least three independent experiments.

It has been shown that Na^+^/K^+^-ATPase can be found in cholesterol enriched membrane microdomains [27] and that Na^+^/K^+^-ATPase-mediated signal transduction is impaired when caveolae is disrupted [28,45]. In order to probe whether Na^+^/K^+^-ATPase is present in lipid rafts in cultures of chick primary myogenic cells, we used the drug methyl-β-cyclodextrin (MβCD) to deplete cholesterol and to disorganize lipid domains. 24 hs-myogenic cells were treated with 10 μM ouabain (for 48 hours) alone, or with 2 mM MβCD (for 30 min) alone, or with 2 mM MβCD for 30 min followed by 10 μM ouabain. 72 hs-myogenic cells were double labeled with an antibody against desmin and with the nuclear dye DAPI. As our group has shown before [4], MβCD alone induced the formation of thicker myotubes as compared to untreated cultures (Figs. 6 and 7f). MβCD alone also induced an increase in the number of nuclei within myotubes (Fig. 7b) and in the total number of nuclei in these cultures (Fig. 7c). Immunofluorescence images show that cholesterol depletion by MβCD did not prevent the 10 μM ouabain induced-effects on myofiber formation (Fig. 6), as confirmed by the quantification of the number of nuclei within myotubes and the thickness of myotubes after treatment of cells with both 2 mM MβCD and 10 μM ouabain (Figs. 7b and 7f). Interestingly, no change in the number of myotubes was observed after treatment of cells with both MβCD and ouabain (Fig. 7d). These results are consistent with the fact that the signaling Na^+^/K^+^-ATPase is not mediating the effect of ouabain in chick myogenic cell cultures. Since we only tested the involvement of lipid rafts in the 24-h step of chick *in vitro* myogenesis, we cannot discard the possibility that Na^+^/K^+^-ATPase could be inserted into lipid rafts in a different stage of chick skeletal muscle differentiation.

Since we found an increase in the number of mononucleated cells together with a decrease in the number of nuclei within multinucleated myotubes after 10 μM ouabain treatment (Fig. 5), we decided to analyze whether this increase in mononucleated cells was related to fibroblasts and/or to myoblasts. Primary chick myogenic cell cultures begin with a population of replicating mononucleated myoblasts and some fibroblastic cells [46]. So, we quantified the number of myoblasts and fibroblasts in untreated and 10 μM ouabain treated cultures and the results show that ouabain treatment induced a 60% increase in the number of fibroblasts and a 20% decrease in the number of myoblasts (Fig. 7e). The decrease in the number of myoblasts (Fig. 7e) is in accordance with the reduction in cell viability found after 10 μM ouabain treatment (Fig. 1). The quantification of the number of myoblasts and fibroblasts was performed by immunolabeling chick myogenic for desmin (a muscle-specific marker) and the nuclear dye DAPI. The nuclei of these two cell types can be easily distinguished in DAPI-stained cultures since fibroblasts have large, flatten and pale nuclei whereas myoblasts have small, round and bright nuclei. We also found a decrease in the number of myoblasts after treatment of cells with MβCD alone or together with 10 μM ouabain (Fig. 7e), but no change in the number of fibroblasts was observed after MβCD treatment. These results are in accordance with previous data from our group showing that MβCD induces an increase in myoblast fusion, a decrease in the number of mononucleated myoblasts and an increase in the formation of multinucleated myotubes [4]. Interestingly, MβCD blocked the proliferative effect of 10 μM ouabain in fibroblast cells, as shown by the decrease in the number of fibroblasts after treatment of cells with MβCD together with 10 μM ouabain (Fig. 7e). It is important to point out that our data show that chick myoblasts are differently affected by ouabain as compared to chick muscle fibroblast cells.

In conclusion, our results show that 10 μM ouabain induce a decrease in cell viability, in the number of myoblasts and in the formation of mature muscle fibers in chick myogenic cells. We also show that the 10 μM ouabain effects in myofiber formation do not involve the MEK-ERK or the p38 signaling pathways, and therefore are probably related to the pump activity function of the Na^+^/K^+^-ATPase. The chick myoblast cell culture model could be used as an important tool for further studies of the molecular and cellular basis of the Na^+^/K^+^-ATPase function in muscle cells.
